# Rolling stones: an instructive case of neonatal cholestasis

**DOI:** 10.1186/s12887-022-03560-3

**Published:** 2022-09-04

**Authors:** Paige Killelea, Shruti Sakhuja, Jose Hernandez, M. John Hicks, Sanjiv Harpavat

**Affiliations:** 1 Department of Pediatrics, Division of Gastroenterology, Hepatology, and Nutrition, Baylor College of Medicine and Texas Children’s Hospital, 6621 Fannin St., Mark Wallace Tower Suite 1010, Houston, TX 77030 USA; 2 Department of Radiology, Baylor College of Medicine and Texas Children’s Hospital, 6621 Fannin St., Mark Wallace Tower Suite 1010, Houston, TX USA; 3 Department of Pathology, Baylor College of Medicine and Texas Children’s Hospital, 6621 Fannin St., Mark Wallace Tower Suite 1010, Houston, TX USA

**Keywords:** Choledocholithiasis, Conjugated bilirubin, Biliary tree, Gallbladder, Case report

## Abstract

**Background:**

Jaundice within the first 1–2 weeks of a neonate’s life will generally self-resolve; however, if it lasts longer than this time frame it warrants further work up. Direct or conjugated hyperbilirubinemia can suggest neonatal cholestasis, which in turn reflects marked reduction in bile secretion and flow. The differential diagnosis for neonatal cholestasis is broad. Neonatal choledocholithiasis is a rare cause of neonatal cholestasis, but should be considered on the differential diagnosis for patients presenting with elevated conjugated bilirubin.

**Case presentation:**

We describe an infant who presented with neonatal cholestasis. He subsequently underwent work up for biliary atresia, as this is one of the more time-sensitive diagnoses that must be made in neonates with conjugated hyperbilirubinemia. He was ultimately found to have choledocholithiasis on magnetic resonance cholangiopancreatography. He was managed conservatively with optimizing nutrition and ursodeoxycholic acid therapy.

**Conclusions:**

We found that conservative management, specifically optimizing nutrition and treating with ursodeoxycholic acid, can be a sufficient approach to facilitating resolution of the choledocholithiasis and conjugated hyperbilirubinemia.

**Supplementary Information:**

The online version contains supplementary material available at 10.1186/s12887-022-03560-3.

## Background

Infants who remain jaundiced after 2 weeks of life must be evaluated quickly [1, 2]. Jaundice caused by indirect (or unconjugated) bilirubin in the newborn period is most commonly due to red blood cell breakdown (hemolysis), breastfeeding or breast milk [3]. Jaundice caused by direct (or conjugated) bilirubin is most commonly due to liver impairment, such as cholestasis. Cholestasis in the newborn can be due to immaturity or impairment of their hepatic excretory function, inborn errors causing dysfunction of the biliary system, as well as an increased susceptibility to viral and toxic insults. In term infants, the most common identifiable causes of neonatal cholestasis are biliary atresia (BA) and rare genetic disorders [2]. In premature infants, it is important to also consider total parenteral nutrition (TPN) use and sepsis [1]. It is important to rule out biliary atresia (BA) immediately as the prognosis is best when interventions are performed early [4]. Here we describe an instructive case of cholestasis caused by bile duct obstruction from gallstones. Management of infants with choledocholithiasis is not well studied, as it is a rare cause of cholestasis in this population. This case demonstrates the importance of considering choledocholithiasis on the differential for cholestasis in neonates.

## Case presentation

**Fig. 1:**
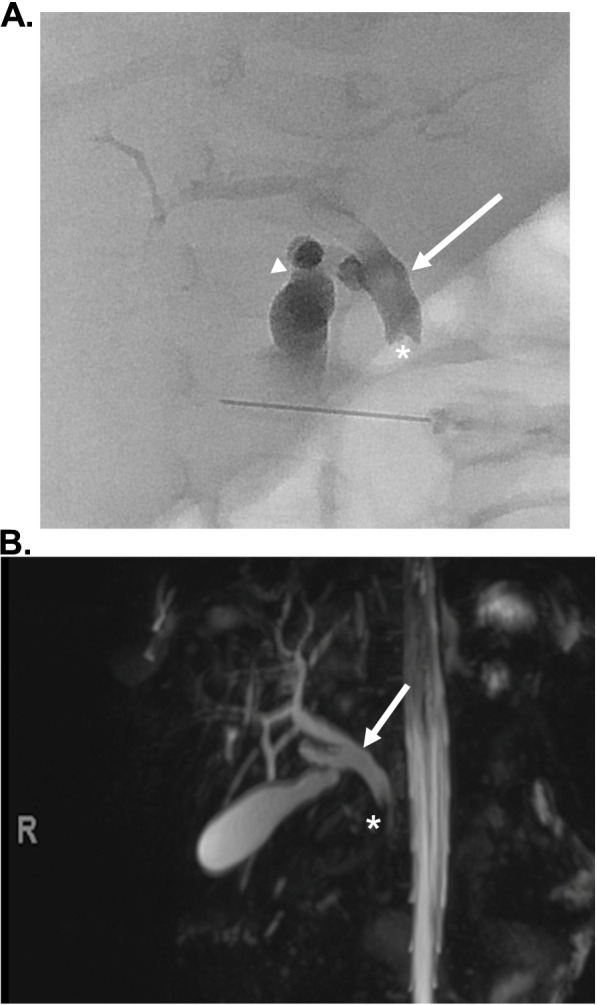
**A** Transhepatic percutaneous cholangiogram obtained at the time of liver biopsy. Arrowhead indicates cystic duct dilation. Arrow indicates CBD dilation. Asterisk marks location of filling defect near the ampulla. **B** Magnetic resonance cholangiopancreatography. Arrow indicates extrahepatic bile duct dilation. Asterisks marks location of theoretical stone

**Fig. 2:**
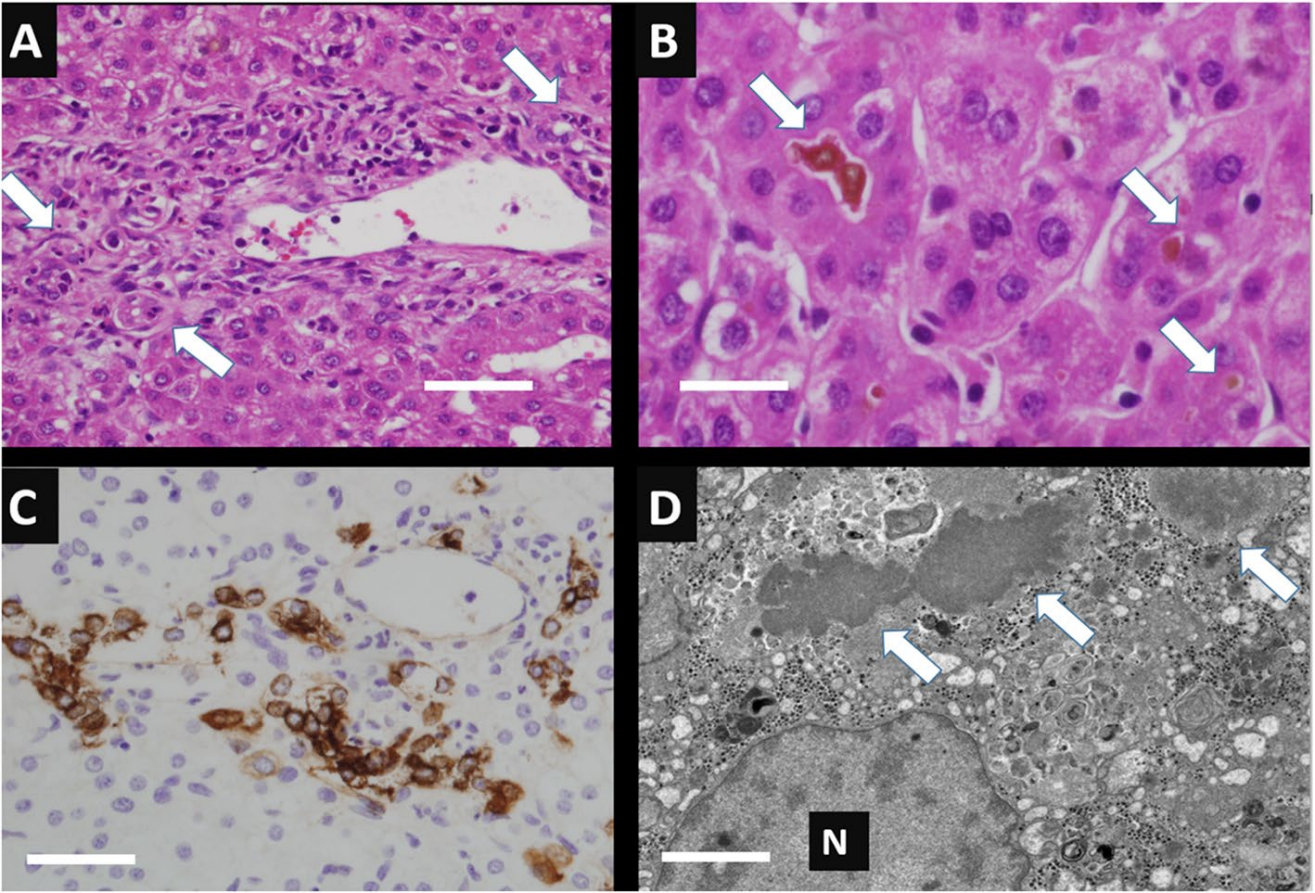
Percutaneous liver biopsy features: **A** Portal regions with mild increase in chronic inflammatory cells with occasional eosinophils and increased number of bile ducts (arrows; scale bar = 100 micrometers; H&E stain, original magnification 400x); **B** Prominent canalicular cholestasis with bile accumulation (arrows; space bar = 40 micrometers; H&E stain, original magnification 1,000x); **C** Bile duct proliferation associated with portal regions highlighted on CK7 immunohistochemical staining (space bar = 100 micrometers; original magnification 400x); **D** Canalicular bile with typical ultrastructural morphology and no features of Byler’s bile (arrows, space bar = 2 micrometer; transmission electron microscopy, original magnification 15,000x)

Our patient is a former 39-week gestational age male who was referred to us by his pediatrician at 2 months of age for 1 week of jaundice and pale stools. He was growing well on formula feeds of Similac Sensitive 6 oz every 3 h until about 2–3 weeks prior to referral when he began to have recurring episodes of emesis. On initial presentation, his weight was appropriate at the 35th% tile (Supplemental Figure). His skin and sclerae were jaundiced, and he had a non-tender, non-distended abdomen. He had conjugated hyperbilirubinemia and elevated liver enzymes (Table 1). Other significant labs included protease inhibitor (PI) typing for alpha-1-antitrypsin deficiency showing heterozygosity for the Z allele (PI*MZ genotype) (Table 2). Initial right upper quadrant ultrasound (US) showed a normal gallbladder, spleen, liver and intra- and extrahepatic bile ducts (no biliary dilation appreciated; common bile duct measured 1.5 mm). Due to the time sensitive nature of diagnosing BA, our patient underwent transhepatic percutaneous cholangiogram and liver biopsy on day 2 of his admission. Cholangiogram showed a dilated common bile duct (CBD) (diameter 6 mm), dilated cystic/intrahepatic bile ducts, and failure of dye flow into the intestines (Fig. 1A). The liver biopsy was consistent with obstruction, showing inflammatory cells, proliferation of bile ducts, and bile accumulation (Fig. 2) (analyzed using Olympus transmitted light microscopy BX60 model with Olympus DP71 camera and Olympus CellSense digital software for capturing images, without downstream processing of images). Polymerase chain reaction (PCR) analysis of liver tissue did not detect parvovirus, adenovirus, human herpesvirus 6 (HHV-6), Epstein Barr virus (EBV), and cytomegalovirus (CMV).

**Table 1:** Laboratory values during evaluation of neonatal cholestasis

	Day 1	Day 5	Day 7	Day 11	Day 13	Day 21
**AST (U/L)**	146 (H)	185 (H)	158 (H)	132 (H)	118 (H)	84 (H)
**ALT (U/L)**	102 (H)	143 (H)	136 (H)	110 (H)	110 (H)	81 (H)
**GGT (U/L)**	415 (H)	597 (H)	603 (H)	631 (H)	611 (H)	464 (H)
**Conjugated bilirubin (mg/dL)**	3.5 (H)	4.8 (H)	4.2 (H)	2.3 (H)	0.4 (H)	0.0
**Unconjugated bilirubin (mg/dL)**	1.5 (H)	1.6 (H)	1.4 (H)	1.0	0.8	0.4

**Table 2:** Other diagnostic laboratory values

PI typing	MZ Type, heterozygosity
**Alpha 1 antitrypsin level**	120 mg/dL
**Bile acids level**	160 umol/L
**TSH**	1.704 mIU/L
**Free T4**	1.2 ng/dL
**Vitamin A level**	0.28 mg/L
**Vitamin E level**	3.2 mg/L
**Vitamin D 25OH level**	5.5 ng/mL (L)
**INR**	0.9
**HHV-6 tissue**	Negative
**EBV tissue**	Negative
**CMV tissue**	Negative
**Genetic cholestasis panel**	SERPINA1(NM_000295.4):c. 1096G > A (p.E366K), heterozygous, pathogenic

Given the extensive ductal dilation and very distal obstruction near the ampulla seen on cholangiogram, we were less concerned about BA and did not proceed to operative exploration or Kasai portoenterostomy. To improve bile flow, the patient was started on ursodeoxycholic acid and the parents were encouraged to continue feedings with his home formula. He remained stable while we continued to monitor daily ALT, AST, GGT and bilirubin. Because his labs remained elevated on day 5, a magnetic resonance cholangiopancreatography was performed to further evaluate the distal obstruction (Fig. 1B). This showed gallstones with upstream biliary dilation and CBD dilation (6 mm). With this new finding of gallstones, we consulted surgery. We ultimately opted for conservative management due to his small size and stable condition. His ursodeoxycholic acid therapy was continued (12 mg/kg twice daily), and we encouraged feeds every 3 hours to stimulate bile flow. We changed his formula to medium chain triglyceride formula to increase fat-soluble vitamin absorption.

On day 6 of admission his stools started to transition from pale to brown. US on day 9 showed a decrease in CBD dilation to 3 mm with no gallstones identified. His conjugated bilirubin continued to downtrend, he continued to demonstrate excellent weight gain on his new formula (Supplemental Figure), and he was ultimately discharged home on day 12 of his stay. Eight days after discharge he had a conjugated bilirubin of 0.0 mg/dL and a follow up US showed normal CBD diameter (1.5 mm).

## Discussion and conclusion

Jaundice develops in 50% of term and 80% of preterm infants, but typically resolves spontaneously within 2–3 weeks [2]. Cholestatic jaundice is caused by a build-up of conjugated bilirubin due to a complete halt or marked reduction in bile secretion and flow [2]. The differential for cholestatic jaundice in a neonate is broad and includes BA, Alagille’s syndrome, alpha 1 antitrypsin deficiency, progressive familial intrahepatic cholestasis, choledochal cysts, cystic fibrosis, metabolic diseases, infection, and sepsis [1].

While choledocholithiasis is common on the differential for cholestasis in adults, choledocholithiasis in a neonate is a rare finding. It is estimated that 0.15–0.22% of children younger than 16 years have cholelithiasis, and that only 10% of these children will have choledocholithiasis. In neonates choledocholithiasis is relatively uncommon, estimated at far less than 1 in 5000 in one study by Yu et al. [5] Neonates most commonly develop gallstones due to hemolysis, ileal disease, congenital anomalies of the biliary tree, hyperalimentation, prolonged fasting, TPN use and sepsis [6]. MZ heterozygosity may also confer an increased risk for gallstone disease for reasons that are not fully understood [7–10]. Some infants with alpha-1-antitrypsin deficiency have poor bile flow so it is possible that heterozygotes also have impaired bile flow which could promote gallstone formation [7–10].

Medical management with ursodeoxycholic acid is recommended in neonates with choledocholithiasis if they are asymptomatic or with mild symptoms. Antibiotics can aid in the resolution of stones in neonates who have signs of infection [5, 11, 12]. In a study of 13 infants with gallstones, 10 remained asymptomatic without surgical management, and stones resolved on their own in 5 of them, with the other 5 either lost to follow-up or with persistent calcified gallstones [6]. In another study, 3 infants with choledocholithiasis treated with antibiotics and ursodeoxycholic acid had resolution of gallstones and CBD dilation [11]. It has been reported that choledocholithiasis resolves spontaneously in up to 35–60% of cases seen in neonates and infants [5].

Invasive management has also been used in management of choledocholithiasis in neonates. In a 3-week-old full-term infant with choledocholithiasis who was not improving with medical management, cholecystectomy and stone extraction was successfully performed [13, 14]. Another study describes a premature infant with choledocholithasis and compromised hepatic function who was successfully treated with cholecystectomy and t-tube placement [13, 14]. In a case report of a 4 month old, ERCP and sphincterotomy were chosen as the route of management because his liver labs were not normalizing [15].

While BA is an important differential diagnosis for cholestasis in infants, this case is a reminder that not all obstruction in neonates is BA. Clues arguing against BA in this case included bile duct dilation (though not seen on initial US) and obstruction only at the most distal biliary segment near the intestines. In BA, bile ducts are not dilated despite extrahepatic bile duct obstruction, perhaps because of inflammatory and/or fibrotic changes around the biliary system [16–18]. In addition, obstruction typically involves more segments of the biliary system, though a minority of cases have obstruction only affecting the CBD [16–18]. Our patient presented with conjugated hyperbilirubinemia and acholic stools and was found to have proliferating bile ducts, which are features of BA. While it is important to evaluate neonates for BA quickly, it is also useful to concurrently consider other causes such as choledocholithiasis which can often be managed with ursodeoxycholic acid therapy and adequate feeds.

In conclusion, this case demonstrates how choledocholithiasis can cause neonatal cholestasis and be treated non-surgically with conservative care. Clinicians should include choledocholithiasis in their differential when evaluating cholestatic infants for BA.

## Supplementary Information

**Additional file 1: Supplemental Figure.** Weight-for-age trend over time

## Data Availability

Data sharing is not applicable to this article as no datasets were generated or analyzed during the current study.

## Abbreviations

BA: Biliary atresia
TPN: Total parenteral nutrition
US: Ultrasound
PI: Protease inhibitor
CBD: Common bile duct
PCR: Polymerase chain reaction
HHV-6: human herpesvirus 6
EBV: Epstein Barr virus
CMV: Cytomegalovirus
