# Supplementary figures and images for: Rolling stones: an instructive case of neonatal cholestasis
Source: BMC Pediatr. 2022 Sep 4;22:526. doi: 10.1186/s12887-022-03560-3 (PMC9441063; doi:10.1186/s12887-022-03560-3)

**Supplemental Figure**


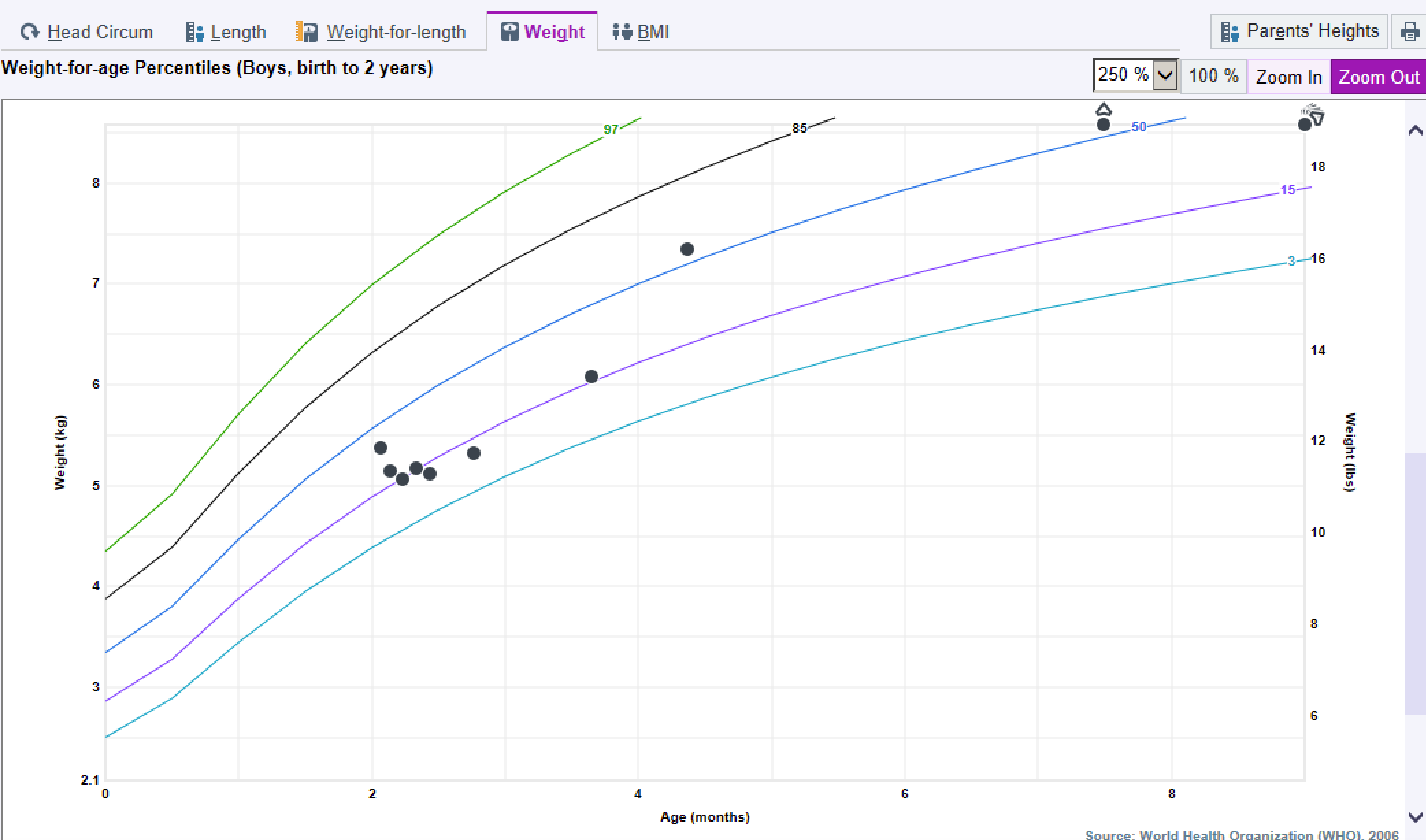

Supplement: Supplementary file 1 — Additional file 1: Supplemental Figure. Weight-for-age trend over time [file 12887_2022_3560_MOESM1_ESM.docx]
